# gH625-liposomes deliver PACAP through a dynamic *in vitro* model of the blood–brain barrier

**DOI:** 10.3389/fphys.2022.932099

**Published:** 2022-08-19

**Authors:** Teresa Barra, Annarita Falanga, Rosa Bellavita, Vincenza Laforgia, Marina Prisco, Stefania Galdiero, Salvatore Valiante

**Affiliations:** ^1^ Deparment of Biology, University of Naples Federico II, Naples, Italy; ^2^ Department of Agricultural Sciences, University of Naples Federico II, Naples, Italy; ^3^ Department of Pharmacy, School of Medicine, University of Naples Federico II, Naples, Italy

**Keywords:** blood–brain barrier, drug delivery, PACAP, liposome, millifluidic bioreactor

## Abstract

The blood–brain barrier (BBB) selectively protects the central nervous system (CNS) from external insults, but its function can represent a limit for the passage of therapeutic molecules. Numerous *in vitro* models of the BBB have been realized in order to study the passage of drugs for neurodegenerative diseases, but these *in vitro* models are not very representative of the physiological conditions because of a limited supply of oxygen and nutrients due to static conditions. To avoid this phenomenon, we used a millifluidic bioreactor model that ensures a circulation of the medium and, therefore, of the nutrients, thanks to the continuous laminar flow. This dynamic model consists of a double-culture chamber separated by a membrane on which brain endothelial cells are cultured in order to evaluate the passage of the drug. Furthermore, in the lower chamber, SH-SY5Y were seeded as 3D spheroids to evaluate the drug passage through these cells. As nanodelivery system, we used liposomes functionalized with viral fusion peptide to evaluate the passage of a neuroprotective agent, pituitary adenylate cyclase-activating polypeptide (PACAP), through the dynamic *in vitro* model of the BBB. We showed that our nanodelivery system, made of functionalized liposomes and loaded with specific molecules, efficiently crosses the *in vitro* fluid-dynamic model of the BBB. Our findings represent an important step for further experimental investigations on PACAP administration as a therapeutic agent by an enhanced drug delivery system. Our results can improve the diffusion of good practice in neuroscience laboratories, helping to spread the 3R rules.

## Introduction

### The blood–brain barrier

The blood–brain barrier (BBB) is an anatomical structure that separates blood from extracellular fluid in the central nervous system (CNS). The BBB can protect the brain from substances that can damage it and allows maintaining a constant brain environment. The passage of drugs through the BBB is very limited due to the presence of tight junctions (TJs) between basement membranes (Sandoval and Witt, 2008). Therefore, the delivery and release of drugs in the brain is a challenging topic and attracts a lot of attention considering the growing number of neurodegenerative diseases such as Alzheimer’s disease (Cummings and Cole, 2002), Parkinson’s disease (Alavijeh et al., 2005), Huntington’s chorea (Wohlfart et al., 2012), and HIV encephalitis (Spindler and Hsu, 2012). The functions of the BBB rely on the cellular components, mainly the specialized endothelial cells forming brain capillaries. These cells act as a continuous and selective physical barrier for hydrophilic substances and are involved in the control of molecular trafficking in the CNS (Abbott et al., 2006). They are held together by TJs, astrocytes, pericytes, and basal membranes (Reese and Karnovsky, 1967; Abbott et al., 2006). Moreover, they express a number of transporters responsible for the regulated exchange of nutrients and toxic products (Abbott et al., 2006), including ATP-binding cassette (ABC) transporters, which are present in a higher percentage than other proteins, providing protection for the BBB and also limiting the transportation of drugs to the brain (Alyautdin et al., 2014).

### Millifluidic tool to recreate a blood–brain barrier *in vitro* model

New advanced techniques have begun to gain fertile ground since animal tests are considered very expensive, take a long time, and can provide unsatisfactory results. Taylor et al., in 2019 estimated, collecting data from 37 countries, that annual laboratory animal use for 2015 ranged from 28 to 100 million (Taylor and Alvarez, 2019). This raises the need to develop useful *in vitro* models to satisfy the 3R approach. The current *in vitro* models are simple, fast and reproducible, ethically acceptable and a wide range of cells, including human ones, can be used. There are several *in vitro* models: “organ-on-chip,” devices that contain an integrated electronic parameter monitoring system, allowing the *in vitro* simulation of the physiology of organs and cells (Pandey et al., 2015), and microfluidic systems that combine microengineering techniques with populations of living cells to mimic the characteristics of the *in vivo* environment (McDonald et al., 2000). The flow is an important factor that modifies several environment and cell features: it increases the turnover of nutrients, induces cytoskeletal reorganization, increase in membrane permeability, and causes greater elongation of endothelial cells (Vozzi et al., 2009; Rouwkema et al., 2011; Ucciferri et al., 2014). Despite this, both models have a variable combination of advantages and disadvantages (e.g., small volumes, limited diffusion of nutrients, physiologically-relevant cell density, difficult to remove sticking air bubbles); this allowed to resort to the use of millifluidic systems, which ensure a high recycling of nutrients, larger volumes, and a physiologically-relevant cell density (Giusti et al., 2014). Among the millifluidic dynamic models, bioreactors, allowing the autonomous growth of cells, are able to provide an adequate environment for cell growth through the modification of chemical-physical parameters (Rouwkema et al., 2011). Cells are maintained with a flow that improves cell proliferation, imposes shear stresses, induces junction formation and establishes polarized tissues. These systems also guarantee the high recycling of nutrients, greater volumes of physiologically-relevant cell density to maintain cell growth and monolayer integrity (Giusti et al., 2014). Pandley et al. have constructed a dynamic *in vitro* BBB (DIV-BBB) model based on the culture of endothelial cells inoculated with astrocytes under flow conditions, capable of developing a phenotype like that of cells *in situ* (Pandey et al., 2015; Bobilya, 2010), instead, with a co-culture model with astrocytes and endothelial cells from pig brains, demonstrated the mechanism of transport through the BBB. This model has also been shown to be promising for the treatment of BBB in neuroinflammatory diseases (Cucullo et al., 2011). Among the millifluidic bioreactor systems, we find the Live Boxes (IVTech) that faithfully recreate the physiological conditions found *in vivo*. Their diameter is 20 mm, they are made of polydimethylsiloxane (PDMS), transparent to allow live imaging analysis, and they are modular, allowing the creation of *in vitro* models of increasing complexity. PDMS is composed using soft lithography techniques to obtain specific micro-channels for cell cultures. An oxygen permeable material such as PDMS is essential for the functioning of these devices. The membrane in which we have seeded our cells is made of polyester (PET): It is protein-binding, low adsorption, and absorption. Its results in biocompatibility, excellent chemical resistance and thermal stability and present a high porosity for transport studies. Moreover, it allows the application of higher flow rates with high oxygen delivery while maintaining acceptable values of shear stress on the upper cell surface (Mattei et al., 2014; Giusti et al., 2017). LiveBox2 (LB2, IVTech, Italy) is a double-flow bioreactor, used to recreate physiological barriers *in vitro* as it has a porous membrane that divides into two independent parts. To allow the flow inside them, the Live Boxes are connected to a peristaltic pump, LiveFlow (IVTech, Italy). Using these bioreactors, we can mimic the anatomical-physiological complexity of the BBB *in vivo*, based on ideal criteria such as reproducibility, easiness of culture, fidelity of the physiological architecture, expression of transporter functions, and response to chemical stimuli. This is mandatory when the development of a reliable *in vitro* BBB model is needed to manage neurodegenerative diseases and to formulate a valid pharmacological approach into the envision of the 3R principles.

### Pituitary adenylate cyclase-activating polypeptide

Pituitary adenylate cyclase-activating polypeptide (PACAP) is the most conserved peptide of the secretin/glucagon superfamily (Vaudry et al., 2000). It exists in two different isoforms: PACAP38 and PACAP27, a C-terminally truncated form cleaved PACAP38 (Rudecki and Gray, 2016; Fang et al., 2020). PACAP38 is the predominant form found in the brain, and PACAP27 constitutes a minority of the total brain PACAP content (Arimura et al., 1991), but both display similar biological activity in terms of adenylyl cyclase (AC) stimulation (Miyata et al., 1990). PACAP exerts neurotrophic actions (Shioda et al., 1994; Yuhara et al., 2001; Erhardt and Sherwood, 2004; Reglodi et al., 2004). In addition, PACAP also has anti-inflammatory properties in dopaminergic SH-SY5Y cells (Brown et al., 2013); it protects neurons from 1-methyl-4-phenyl-1,2,3,6-tetrahydropyridine (MPTP) neurotoxic effects in animal models of Parkinson’s disease (Deguil et al., 2010), leading to recovery of dopaminergic function (Takei et al., 1998; Reglodi et al., 2004; Somogyvari-Vighet and Reglodi, 2004; Wang et al., 2008). A synthetic PACAP analog was used to restore tyrosine hydroxylase expression in the substantia nigra and to modulate the inflammatory response in mice (Lamine et al., 2015). PACAP has several beneficial effects in some pathological processes due to its neuroprotective and neurotrophic action (Harmar et al., 1998; Vaudry et al., 2009). Detailed analyses showed that PACAP levels were reduced in the human entorhinal cortex, mid-temporal, superior frontal, and 16 primary visual cortex at both the mRNA and protein levels associated with pathological signs of Alzheimer’s disease; PACAP levels were reduced more in the amyloid plaque but not in the primary visual cortex, a region spared in most cases of the disease (Wu et al., 2006). PACAP is also reduced in several dementias, representing a link between its reduction and age-related (Han et al., 2014). The action on the signaling pathways and therefore the physiological effects depend on the G protein coupled receptors expressed in the different tissues: PAC1-R, VPAC_1_, and VPAC_2_ (Vaudry et al., 2009). In the basal ganglia of Parkinsonian macaque monkey, an important and specific decline of the PACAP receptor was observed in the pathway PACAP/PAC1-R (Feher et al., 2018). PAC1 is mainly expressed in brain regions along with PACAP distribution, while VPAC_1_ and VPAC_2_ are mainly expressed in regions outside of the brain. PAC1-R is, therefore, the principal receptor of PACAP in the CNS (Vaudry et al., 2009; Reglodi et al., 2018; Fang et al., 2020). Amin and Schitz in 2018 showed PACAP transport across the BBB. In preclinical studies, PACAP 38 acts as both an influx (blood to brain) and an efflux (brain to blood) by the protein transport system-6 (PTS-6) in the endothelium. Other studies conducted on PACAP38 on the cerebral arteries show that PACAP38 crosses the endothelium in less amounts to activate receptors in the arterial walls (Dogrukol-Ak et al., 2004; Amin et al., 2012; Amin et al., 2014). Based on the literature, PACAP38 crosses the BBB in a saturable carrier mediated mechanism, instead PACAP27 via transmembrane diffusion from blood to brain (Amin and Schytz, 2018), in efflux via saturable carrier beta-F1 ATPase (Dogrukol-Ak et al., 2004). PACAP stimulates cAMP/PKA- (cyclic adenosine monophosphate/protein kinase A) mediated signaling, and other pathways like PI3K-pathways (phosphoinositol 3 kinase) or calcium-regulated mechanisms involved in repair and neuronal protection. However, PACAP, as an intravenous therapeutic agent, presents some difficulties, such as rapid degradation in the blood and low bioavailability (within minutes). PACAP27 and PACAP38 can interact with phospholipid bilayers at physiological concentrations. Given the particularity of interacting with the phospholipid and self-aggregate systems, it is possible to introduce them into constructs to increase their therapeutic activity (Krishnadas et al., 2003). To improve PACAP stability, many strategies have been adopted like cyclization, N-methylation, lipidation, and PEGylation (Erak et al., 2018). According to the literature , PACAP27 can cross the BBB and its concentration in blood does not decrease as its permeability decreases, unlike PACAP38 (Banks et al., 1993). PACAP27 is relatively resistant to degradation in human plasma *in vitro*, whereas the 38-residue isoform displays a half-life of less than 5 min in isolated human plasma. Bourgault et al. (2008) and Vaudry et al. (2009) suggested that the 28-to-38 region is important for the degradation of PACAP by plasma endopeptidases. A useful approach for the targeted administration of PACAP to the CNS involves the use of liposomes as cargo, modified with amphipathic peptides capable of crossing biological membranes and transporting small molecules and proteins. Cell-penetrating peptides can be used to enhance drug delivery of many macromolecules *in vitro* and *in vivo* (Drin et al., 2003; Copolovici, et al., 2014; Lajoie and Shusta, 2015). Moreover, these peptides transport cargo without degradation of them immediately used in the cytosol (Falanga et al., 2011). The gH625 peptide (Galdiero et al., 2005), which is a disturbing domain of the membrane derived from the *Herpes simplex* type 1 virus, has suitable features in this regard. This peptide promotes vesicular fusion (Falanga et al., 2011). gH625 enters in SH-SY5Y cells and U-87MG glioblastoma cells *in vitro* and can efficiently cross the rat BBB *in vivo* labelling cell neurites, showing no toxic effects (Valiante et al., 2015). It has also been demonstrated that the intravenous administration of gH625-liposomes loaded with rhodaminated PACAP27 permits its release across the rat BBB (Iachetta et al., 2019). Based on this background, the aim of this study is to evaluate the ability of gH625-liposomes to deliver PACAP27 through a dynamic *in vitro* model of BBB. The *in vitro* model of the BBB consists of endothelial brain cells (bEnd.3) seeded in an upper chamber and the human 3D cell lines SH-SY5Y (neuroblastoma cells) cultured in the lower chamber of the millifluidic bioreactor (Livebox2, LB2).

## Materials and methods

### Peptide synthesis

The gH625 with the cysteine at C-terminal (Ac-HGLASTLTRWAHYNALIRAF-Cys)-and PACAP27 (HSDGIFTDSYSRYRKQMAVKKYLAAVL-CONH2) peptides were synthesized 17 using a standard Fmoc solid-phase (GL Bio chem Ltd., Shanghai, China) as previously reported (Iachetta et al., 2019) and were obtained with good yields of about 40%. PACAP27 was labeled on resin with rhodamine (5(6)-Carboxytetramethylrhodamine N-succinimidyl ester) for fluorescence measurements, as reported (Rapaport and Shai, 1991). Peptides were fully deprotected and cleaved from the resin with an acid solution of trifluoroacetic acid and scavengers. The crude peptides were precipitated with ice-cold ethyl ether, and purified by preparative reverse-phase HPLC with a solvent mixture of H_2_O and 0.1% trifluoroacetic acid (solvent A) and CH_3_CN and 0.1% trifluoroacetic acid (solvent B), with a linear gradient B over 20 min at a flow rate of 15 ml/min. Peptide identity was confirmed using an LTQ-XL Thermo Scientific linear ion trap mass spectrometer. For the synthesis of DSPE-PEG2000-gH625, 1 eq of DSPE-PEG2000-Mal was reacted with 1 eq of pure gH625-Cys in DMF in the presence of 5 eq of triethylamine (5 eq) for 24 h. The reaction was monitored by RP-HPLC, when completed the solvent was evaporated and the product was analyzed using an LTQ-XL. 1,2-Dipalmitoyl-snglycero-3-phosphocholine (DPPE), cholesterol, 1,2-distearoyl-sn-glycero-3 phosphoethanolamine-N- [maleimide (polyethylene glycol)-2000] (DSPE-PEG2000-Mal) were purchased from Avanti Polar Lipids (Birmingham, AL, United States). Coupling reagents, N,Ndiisopropylethylamine (DIEA), piperidine, trifluoroacetic acid (TFA), Rhodamine (5 (6)- Carboxytetramethylrhodamine N-succinimidyl ester, and Rink amide resin (0.62 mmol/g of loading substitution), were purchased from Iris-Biotech GMBH.

### Liposome preparations

Large unilamellar vesicles (LUVs) consisting of DPPC/Chol (70/30 mol/mol) were prepared as previously reported (Galdiero et al., 2005). Furthermore lipids, DSPE-PEG2000-gH625, and PACAP -Rho were dissolved in chloroform, the solvent was removed with a nitrogen gas stream, and the sample was lyophilized overnight to obtain a lipid film. The obtained film was suspended in buffer to produce LUVs, freeze-thawed eight times, and then extruded 10 times through polycarbonate membranes with 0.1 μm diameter pores (Northern Lipids). The hydrodynamic diameters (DHs) and polydispersity index (PDI) of PACAP-Rho loaded liposomes (Lipo) and PACAP-Rho loaded gH625-liposomes (gH625-Lipo) were measured using dynamic light scattering (DLS) (Malvern Zetasizer Nano ZS, Malven, United Kingdom). The analysis was performed with an He–Ne laser of 4 mW operating at 633 nm at a scattering angle fixed at 173° and at 25°,respectively.

### bEnd.3 cell culture

Murine endothelial brain cells (bEnd.3) were grown in Dulbecco’s Modified Eagle’s Medium High glucose, supplemented with fetal bovine serum (10%, Sigma-Aldrich-Saint Louis, United States), penicillin/streptomycin (100 U/ml, Sigma-Aldrich-Saint Louis, United States), L-glutammine (2 mM, Sigma-Aldrich-Saint Louis, United States), and gentamycin (40 μg/ml, Sigma-Aldrich-Saint Louis, MO, United States) at 37°C, 5% CO_2_ in a humidified incubator. Cells grow adherent in 25 cm^2^ flasks. The medium was changed twice a week. When 70% confluent, cells were enzymatically detached with trypsin-EDTA (Sigma-Aldrich-Saint Louis, United States).

### Lucifer yellow assay

**FIGURE 1:**
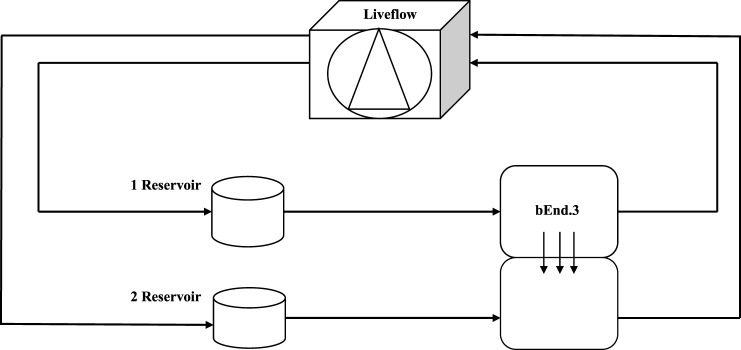
Schematic view of a dynamic bioreactor: Live Box 2 (LB2) is composed of an upper chamber connected to a medium reservoir and to a Liveflow pump (250 μL/min) and a lower chamber connected to a medium reservoir and to a Liveflow pump (250 μL/min). In the upper chamber, bEnd.3 cells are seeded on the porous membrane on the holder.

Lucifer yellow (LY) is a hydrophilic dye and its fluorescence can be used to determine the permeability coefficient (Pc) of the cerebral endothelial monolayer, thus the barrier integrity (Zhao et al., 2019). In *in vitro* BBB models, Pc values are considered in the order of 10–4 cm/min for different solutes (Cecchelli et al., 2014; Deli et al., 2005; Reichel et al., 2003; Wolff et al., 2015). Moreover, LY has a Stokes shift of about 108 nm, compared to other small Stoke shifts (Ren et al., 2018). This allows one to determine a spectral separation such as to provide enough fluorescence data to determine cell permeability. Our experiments are carried out using a dynamic millifluidic tool called Livebox2 (LB2, IVTech, Italy) made of polydimethylsiloxane (PDMS), commonly used to mimic physiological barriers (Giusti et al., 2014). This bioreactor is composed of two parts: a lower chamber and an upper chamber, each with an independent flow, divided by a porous membrane (ipPORE, Belgium). These membranes, thanks to their porosity (0.45 µm), allow the passage of nutrients but prevent the passage of cells, causing easy adhesion and reducing the binding of non-specific molecules. The chambers are also connected to a peristaltic pump circuit (Liveflow, IVTech, Italy), which allows a continuous recycling of nutrients and allows to evaluate the diffusion of drugs between the two chambers. The cells seeded on the porous membrane are bEnd.3 (murine endothelioma) commonly used for BBB *in vitro* (Dos Santos Rodrigues et al., 2019). This membrane was first adapted to 80% ethanol for 15 min. Then, the wet membrane is laid on the lower part of the holder. Once assembled, the holder is placed in 6-well plates covered with a complete DMEM culture medium for 24 h. After 24 h, the LB2 culture chamber is assembled with the holder containing the membrane, in the central portion between the upper and lower chamber (Figure 1). Later, 1 ml of DMEM complete medium was injected into the lower chamber and 150,000 bEnd.3 cells in 100 µL of DMEM complete medium were seeded onto the membrane from the inlet tube of the upper chamber, adding 500 µL of complete DMEM to make the cells in the inlet tube flow. The LB2 was then placed in an incubator for a week at 37° and 5% of CO_2_. To adapt the cells to the flow, the LB2 chamber was connected to the Liveflow. The circuit was first connected to the mixing chambers containing 8 ml of DMEM complete medium, and then LB2 was connected to the LiveFlow at a nominal flow of 250 µL/min. In the mixing chambers, medium was changed twice a week. Permeability measurements were carried out on different days: 1, 3, 6, and 8. In these different days, the growth medium was removed, and we added 1 ml of pre-warmed (37°C) transport buffer (25 mM HEPES, 145 mM NaCl, 3 mM KCl, 1 mM CaCl2, 0.5 mM MgCl2, 1 mM NaH2PO4, and 5 mM glucose, pH 7.4) to the basolateral side (Zhao et al., 2019). In the apical side of the porous membrane, 20 µM of LY (Sigma-Aldrich-Saint Louis, United States) was added and incubated at 37°C for 60 min. Then, 30 µL of the LY sample was removed from each apical compartment and transferred into tubes, diluting the sample using 10-fold transport buffer. After removing 500 µL from the basolateral compartment, a series of LY standards have been prepared. Each standard was performed in duplicate on a black 96-well plate. Fluorescence has been measured using a Synergy HTX Multi-mode microplate reader (Ex: 428 nm, Em: 536 nm). Permeation coefficient (Pc) was calculated from the following equation:

$$\mathrm{Pc}\;=\;\frac{\mathrm{Vb}\;\;}{\mathrm{Ca}\;x\;A\;x\;\frac{\mathrm{cb}}{T}}$$

where Vb is the volume of the lower chamber (1 ml), Cb is the concentration of LY (μM) in the lower chamber, Ca is the concentration of LY (μM) in the upper chamber, A is the membrane area (1.12 cm^2^) (Giusti et al., 2014; Zhao et al., 2019), and T is the time of transport (3600 s). This test was useful to determine when bEnd3 cells form the tightest monolayer for maximum barrier integrity.

### Immunofluorescence assay

The Lucifer yellow assay indicates us the formation of a stable and integral barrier to day 6–8. After a week, different indirect immunofluorescence assays were performed to evaluate protein junction formation on bEnd.3 cells cultured on the porous membrane of LB2 connected to LiveFlow at a nominal flow of 250 µL/min. After 7 days, cells were washed with phosphate buffered saline (PBS) and fixed with a 4% paraformaldehyde solution (PFA) for 15 min. PFA was subsequently saturated by incubation with 0.1 M glycine. Cells were then permeabilized with PBS 0.4% Triton X 100 followed by another permeabilization with PBS 0.1% Triton X 100. Blocking was performed using 4% BSA for 30 min followed by incubation with the antibodies: Anti-ZO1 tight junction proteins (3 µg/ml in 1%BSA/PBS, Abcam, Cambridge, United Kingdom), Anti-N-cadherin adherens junction proteins, (1:100 in 1% BSA/PBS, Santacruz biotechnology, California, United States), Anti-β catenin adherens junction proteins (1:100 in 1%BSA/PBS, Abcam, Cambridge, United Kingdom) overnight at 4°C. After 24 h, cells were washed three times with PBS, and then incubated with AlexaFluor 488 (1:200, Cambridge, United Kingdom) secondary antibody for 1 h. Nuclei were stained with Höechst 33258 (1:1000, Invitrogen, Carlsbad, United States) for 15 min at room temperature. Cells were acquired with an Axioskop epifluorescence microscope (Carl Zeiss) with a ×40 objective, using the filter for the Höechst 33258 (ex: 360 nm, em: 452 nm) and that for Alexa Fluor 488 (ex: 488 nm, em: 530 nm), an Axiocam MRc5 camera (Carl Zeiss) and Axiovision 4.7 software (Carl Zeiss). Similar cellular fields were chosen for different experimental groups. For each experimental condition three immunofluorescences were repeated and were randomly chosen different fields for data analysis. For each experimental condition three immunofluorescences were repeated and were randomly chosen different fields for data analysis. The acquired images were corrected for brightness and contrast through Fiji software.

### Lactate dehydrogenase assay

Lactate dehydrogenase (LDH) assay (ThermoFisher Scientific, Massachusetts, United States) was used to test bEnd.3 cells’ cytotoxicity on the porous membrane of LB2. It is a colorimetric method used to quantify cellular cytotoxicity. Damaged plasma membrane releases lactate dehydrogenase (LDH), a cytosolic enzyme found in several types of cells. LDH catalyzes the conversion of lactate to pyruvate via reduced NAD + to NADH. Diaphorase then uses NADH to reduce the tetrazolium to formazan salt, which can be measured at 490 nm. The amount of formazan is proportional to the amount of LDH released into the medium, indicative of cytotoxicity. To perform the LDH assay, after 7 days, bEnd.3 cells are lysed with a 1 mM PBS/EDTA solution, and then transferred to a 96-well plate. Subsequently, the spontaneous and maximum LDH activity is measured: 50 µL of all the samples are transferred to a new 96-well plate to which 50 µL of reaction mixture will be added. After incubating for 30 min in the dark, 50 µL of stop solution will be added to each well. Non-viable cells convert the tetrazolium salts into formazan red. To measure the absorbance, expressed in optical density (O.D), a spectrophotometric reading was carried out at 490 nm, using a plate reader (Synergy HTX Multi-mode microplate reader). The absorbance of this compound is directly proportional to the amount of LDH released by the cells. Three assays were performed, and for each experimental class, the test was performed in triplicate.

### Spectrofluorimetry assay

A spectrofluorimetry assay was performed to evaluate the ability of gH-625 liposome compared to only liposome to deliver PACAP through a bEnd.3 monolayer in a millifluidic bioreactor (LB2). After 7 days of bEnd.3 culture in LB2, liposomes were injected into the upper chamber, in correspondence of the membrane, with PACAP-Rho 20 µM (gH625-lipoPACAP-Rho). To evaluate the passage of the gH625-lipoPACAP-Rho in the lower chamber, a spectrofluorimetric experiment was performed on samples of medium taken at regular intervals (30, 60, 90, and 120 min). After 30 min of the injection of the gH625-lipoPACAP-Rho in the upper chamber, the supernatant (100 µL) was taken from the outlet tube of the upper chamber and from the outlet tube of the lower chamber and placed in a 96-well plate. The control group was obtained with liposome-PACAP-Rho. This solution was read at 540–580 nm fluorescence with an Infinite 200M spectrophotometer (TECAN). Each spectrofluorimetric assay was performed in triplicate.

### SH-SY5Y cell culture

Neuroblastoma cell lines enriched to the neural portion (SH-SY5Y-N) were grown in Dulbecco’s Modified Eagle’s Medium High glucose, supplemented with fetal bovine serum (10%, Sigma Aldrich-Saint Louis, United States), penicillin/streptomycin (100 U/ml, Sigma-Aldrich-Saint Louis, United States), L-glutammine (2 mM, Sigma-Aldrich-Saint Louis, United States), and gentamycin (40 μg/ml, Sigma Aldrich-Saint Louis, MO, United States) at 37°C with 5% CO_2_ in a humidified incubator. Cells grow adherent in 25 cm^2^ flasks. Medium was changed twice a week. When 70% confluent, cells were enzymatically detached with trypsin-EDTA (Sigma-Aldrich-Saint Louis, United States).

### Pituitary adenylate cyclase-activating polypeptide ELISA assay

An ELISA assay (Mybiosource, San Diego, United States) was performed to evaluate the amount of PACAP released by gH625-liposome. bEnd.3 cells were cultured in LB2 connected to Livebox1 (LB1, IVTech, Italy), containing SH-SY5Y differentiated in dopaminergic neuron by retinoic acid 10 μM. LB1 is a bioreactor with only one chamber connected to a single mixing chamber. This bioreactor is useful for the preliminary experiments to set the suitable flow that does not cause shear-stress to the cells. The mixing chambers of both Liveboxes contained 8 ml of DMEM culture medium. Once the cells were adapted to the Liveflow (250 µL/min), in the upper mixing chamber of LB2, liposomes were injected with PACAP (gH625-lipoPACAP) at a concentration of PACAP of 20 µM. Samples of medium were taken at regular intervals (30, 60, 90, and 120 min). Specifically, after 30 min, the supernatant (100 µL) was taken from the outlet tube of the upper chamber, from the outlet tube of the lower chamber of the LB2, and from the outlet tube of the LB1. Finally, 1 ml of samples were collected from the upper and lower mixing chambers of LB2 and from the mixing chamber of the LB1. An indirect ELISA protocol was performed using standards from 0 to 1000 pg/ml. To measure the absorbance, expressed in optical density (O.D.), a spectrophotometric reading was performed at 450 nm using an ELISA plate reader (Thermo electron company-Multiskan Ascent).

### 3D SH-SY5Y cells cultured in LB1

The hanging drop method was used for the three-dimensional structures of SH-SY5Y cells. This technique has proved to be useful in hepatocytes and in engineering cardiac spheroids (Chitnis and Weiner, 2017; Polonchuk et al., 2017; Shri et al., 2017) and to study toxicity and in the study of BBB for the employment of neurotoxicity (Nzou et al., 2018). This method exploits the ability of cell-cell and cell-extracellular matrix cohesion (Foty, 2011) within a hydration chamber in which, thanks to the force of gravity, the cells, in direct contact with each other, form aggregates. SH-SY5 cells enriched to the neural portion, are deposited in “drops” inside the lid of a 60 mm dish. The drops must be spaced apart to allow for the formation of the individual spheroids. A total of 10 ml of PBS was placed on the bottom of the dish to create a hydration chamber to favor the formation of aggregates. Once the drops had been sown, the lid of the dish was turned upside down on the bottom containing PBS and placed in an incubator at 37°C, with 5% CO_2_ and humidity controlled for 48 h. After 48 h, the formation of the aggregates was checked with the help of a stereomicroscope and these cells were transferred to LB1 connected to a Liveflow and to their respective mixing chamber with a DMEM cell medium to set the nominal flow suitable to not create shear-stress conditions for the spheroids. The flow chosen was 150 μL/min for 24 h.

### 3D SH-SY5Y immunofluorescence assay

Different indirect immunofluorescence assays were performed in 3D SH-SY5Y cells in dynamic culture to assess the presence of Ki-67 proliferation protein, ZO-1 tight junction protein, and β3-tubulin neural marker. After 24 h of flow connection, spheroids were fixed with cold methanol for 15 min and then washed with PBS to eliminate all fixative residues. Non-specific site blocking was performed with 3% BSA (bovine serum albumin) in 0.1% Triton-PBS for 30 min. Incubation with primary antibodies in 1% BSA/PBS were performed for 1:45 min. The antibodies used (1% BSA/PBS) were: Anti-Ki-67, Anti ZO-1, and Anti-β3 tubulin (Abcam, Cambridge, United Kingdom). Subsequently, after three washes in 0.1% Triton-PBS, cells were incubated for 1 h with the secondary antibodies: AlexaFluor 488 (1:500 in 1% BSA/PBS, Invitrogen, Carlsbad, United States) and Alexafluor 594 (1:500 in 1% BSA/PBS, Invitrogen, Carlsbad, United States). Cell nuclei were labeled with DAPI (1:1000 in PBS, Invitrogen, Carlsbad, United States) for 5 min. Images were acquired with the JuLi ™ Stage_RealTime Cell History Recorder microscope with ×10 objective, using three different channels: DAPI, RFP, and GFP. For each experimental condition, three immunofluorescence assays were repeated, and different fields were randomly selected for data analysis. The captured images were corrected for brightness and contrast using Fiji software.

### gH625-lipoPACAP-Rho through a 3D dynamic *in vitro* blood–brain barrier millifluidic model

Once the 3D SH-SY5Y cells adapted to the flow, after 24 h, they were transferred from the LB1 to the lower chamber of the LB2 containing bEnd.3 cells were seeded in the upper chamber for 7 days to perform the passage of gH625 functionalized liposomes loaded with rhodaminated PACAP (gH625-lipoPACAP-Rho) in a 3D dynamic *in vitro* BBB millifluidic model (Figure 11). LB2 was connected to the respective mixing chamber and to a peristaltic pump, and the flow was then set at 150 μL/min for 24 h. After 24 h, gH625-lipoPACAP-Rho was injected into the upper chamber, loaded with 20 µM PACAP-Rho.

## Results

### Liposome characterization

Liposomes loaded with PACAP-Rho and functionalized on their surface with gH625 were characterized using dynamic light scattering (DLS). The hydrodynamic diameters (DHs) and polydispersity index (PDI) of all liposomes were measured, three independent experiments were performed for each sample, and each measurement was performed at least in triplicate. Lipo-PACAP and gH625-lipoPACAP present a polydispersity index (PDI) < 0.3 indicating a good size distribution (Table 1).

**TABLE 1:** Results of the polydispersity index (PDI) for lipo-PACAP and gH625-lipoPACAP is < 0.3. This represents a good size distribution.

Sample	Mean diameter (nm)	Mean PDI
lipo-PACAP	151.2±1.274	0.080±0.010
gH625-lipoPACAP	193.9±8.050	0.265±0.025

### Lucifer yellow assay

**FIGURE 2:**
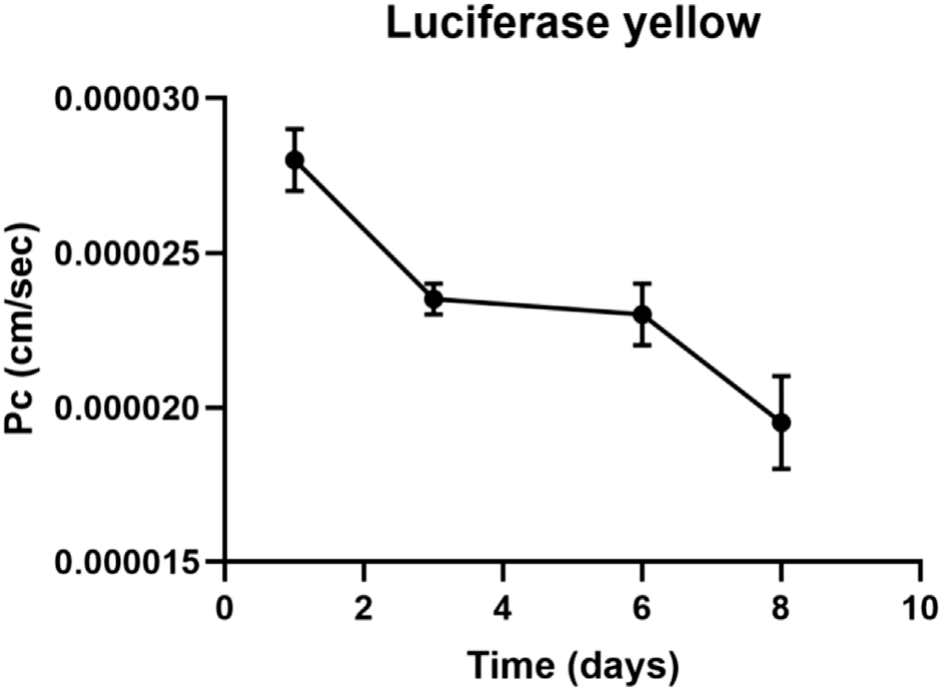
Luciferase yellow permeation through bEnd.3 monolayer cells. LY permeation decreases until day four, where it remains constant until day six to decrease until day eight. LY permeation change indicates the formation of tight junction.

**FIGURE 3:**
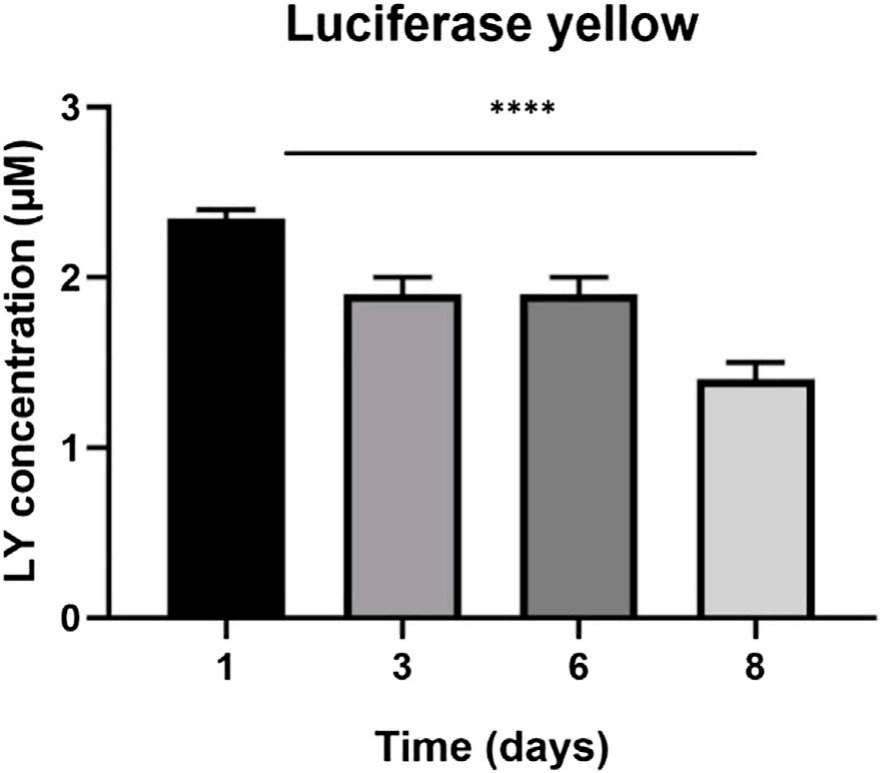
Luciferase yellow concentration (µM) across eight days. The graph shows the means ± SEM of three experiments. Statistical analysis was performed through the analysis of variance (ANOVA) and Dunnet’s posttest. The differences were considered significant compared to day one (1). ***p < 0.001.

The LY added in the apical side is expected to traverse the intercellular tight junctions and accumulate in the basolateral side. Greater concentrations of LY in the basolateral side indicate an immature, not-fully functional barrier, while lower concentrations reflect restricted transport due to the presence of functional TJs, resulting in a mature barrier. LY permeation decreases until day four, where it remains constant until day six, then decreases until day eight. Also luciferase yellow concentration (µM) decreased in 8 days (Figures 2, 3).

### Immunofluorescence assay

**FIGURE 4:**
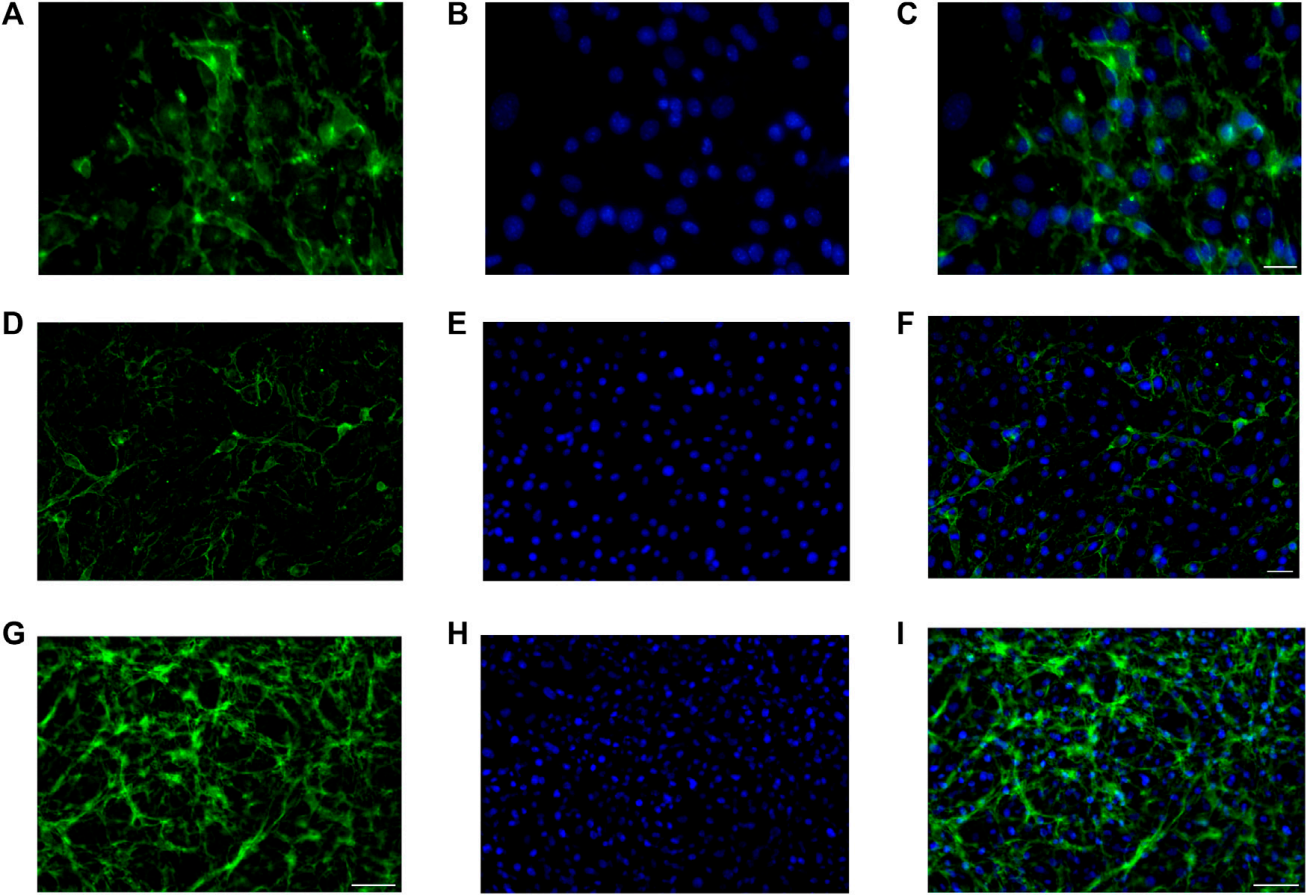
Immunofluorescence for ZO1, GFP **(A**–**C)**, N-cadherin, GFP **(D**–**F)**, β-catenin, and GFP **(G**–**I)** on bEnd.3 cultured in the LiveBox2 fluid dynamic bio incubator. Secondary antibody, GFP emission. Nuclei were labeled with Höechst 33258. Scale bars were: 50 µm **(C)**; 20 µm **(F)**; and 100 µm **(I)**.

Anti ZO-1, anti N-cadherin, and anti-β catenin immunofluorescence for bEnd.3 cells was performed to evaluate the formation of the barrier and its integrity. bEnd.3 cells were seeded on the porous membrane of LB2 and after 1 week of culture, to encourage the formation of junctions, different indirect immunofluorescence assays were performed. After 24 h of the antibodies’ incubation, bEnd.3 cells showed an evident fluorescence signal for all three junction proteins (Figure 4). Nuclei were stained with Höechst 33258.

### LDH assay

**FIGURE 5:**
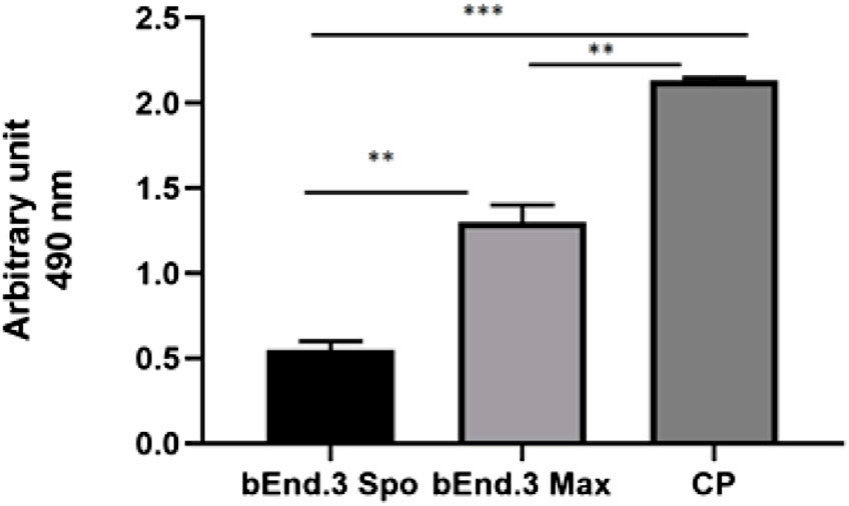
LDH assay for bEnd.3 cells after seeding for eight days. Citotoxicity is less in the bEnd.3 spontaneous activity (bEnd.3 Spo) than in the bEnd.3 maximum activity (bEnd.3 Max) compared to the positive control (PC) at 490 nm. The graph shows the means ± SEM of three experiments. Statistical analysis was performed through the analysis of variance (ANOVA) and Dunnet’s posttest. The differences were considered significant compared to positive c(PC). ***p < 0.0001; **p < 0005.

An LDH assay test was used to evaluate the cytotoxicity of the bEnd.3 cells seeded on the porous membrane of an LB2 bioreactor after 7 days. The graph shows an increase in cytotoxicity in the bEnd.3 maximum activity rather than in the bEnd.3 spontaneous activity compared to the positive control (Figure 5).

### Spectrofluorimetry assay

**FIGURE 6:**
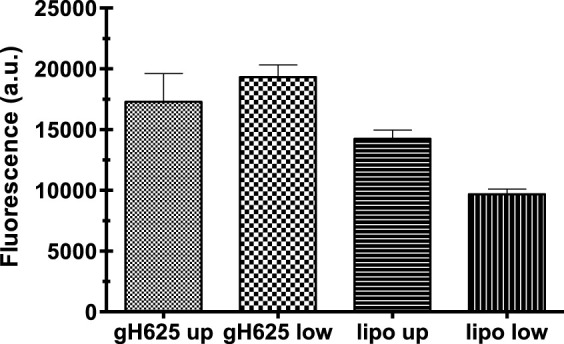
Spectrofluorometric analysis of rhodaminated PACAP (PACAP-Rho) delivery across the BBB dynamic *in vitro* model. Functionalized liposomes and non-functionalized liposomes were loaded with PACAP-Rho and injected in the upper flow. The passage beyond the endothelial cell layer was then evaluated by sampling downstream the upper flow and the lower flow, respectively. Dati ±SEM.

**FIGURE 7:**
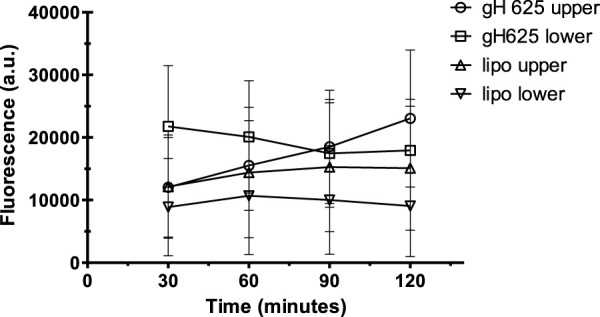
Spectrofluorometric analysis of rhodaminated PACAP (PACAP-Rho) delivery across the BBB dynamic *in vitro* model. After 30 min the fluorescence of gh625-lipoPACAP-Rho is higher in the lower chamber of LB2 compared to lipoPACAP-Rho.

**FIGURE 8:**
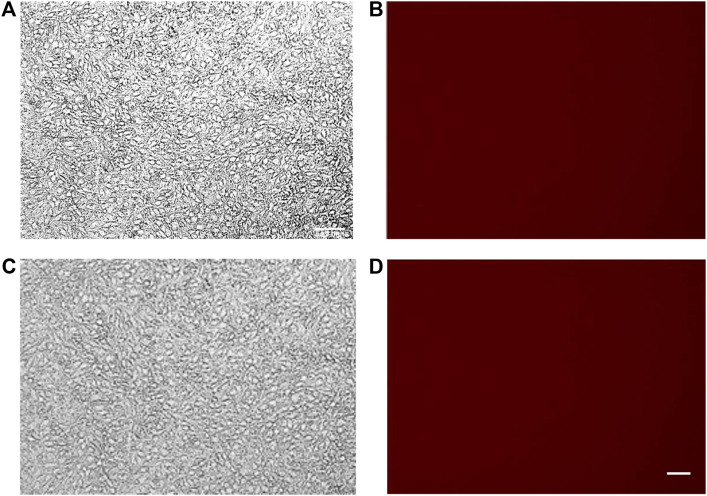
bEnd.3 before **(A**,**B)** and after **(C**,**D)** PACAP loaded functionalized liposome passage. bEnd.3 seeded in the LiveBox2 bio incubator do not retain red fluorescence (no PACAP-Rho) indicating that PACAP-Rho loaded functionalized liposomes pass the bEnd3 endothelial layer and become available in the LiveBox2 bottom chamber. The scale bar was 20 µm.

Spectrofluorimetry was performed to evaluate the passage of PACAP-Rho mediated by gH625-liposome through the porous membrane in an LB2 bioreactor containing bEnd.3 cells. After 30 min of the injection of gH625-lipoPACAP-Rho in the inlet of the upper chamber, samples of medium were taken at regular intervals (30, 60, 90, and 120 min) in the outlet of both the cameras (up and low). There was an increase of PACAP-Rho fluorescence in the lower chamber compared to the upper camera after 30 min of injection (Figure 6), and the fluorescence of the PACAP-Rho bound to gH625-liposome remained high for 2h of the experiment (Figure 7). This increase is more consistent than the non-functionalized liposome loaded with PACAP-Rho. The LB2 containing bEnd.3 cells were then placed in the JuLi ™ Stage Real-Time Cell History Recorder microscope to continuously acquire images of the cells seeded on the porous membrane before and after the passage of the gh625-liposomePACAP-Rho. The images show an absence of signal for PACAP-Rho on bEnd.3 cells, demonstrating the passage of this latter in the lower chamber. Moreover, cells do not appear to be morphologically damaged by the passage of the gH625-liposomePACAP-Rho (Figure 8).

### Pituitary adenylate cyclase-activating polypeptide ELISA assay

**FIGURE 9:**
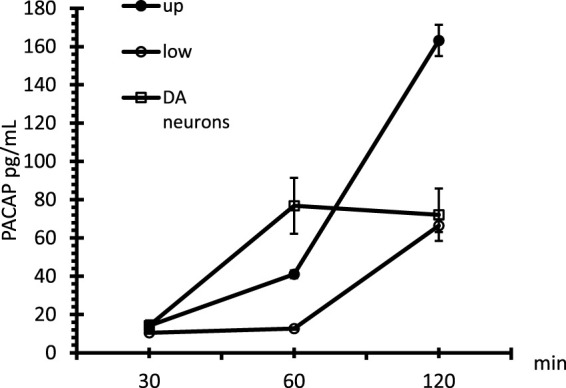
Time course of PACAP in the microfluidic millifluidic system, assessed by the PACAP Elisa assay. Up = upper chamber, low = lower chamber, DA neurons = dopaminergic neurons. Data are expressed as ±SEM.

Finally, once we determined the different passages between gH625-lipoPACAP-Rho and lipoPACAP-Rho, we performed the time course of the PACAP Elisa assay only for gH625 liposomes. We demonstrated an increase of PACAP concentration endothelial cell chambers during time after injection of gH625-lipoPACAP in the endothelial cell compartment . SH-SY5Y dopaminergic neurons are exposed to the full concentration (i.e., about 10^−8^ M) of PACAP within 60 min (Figure 9).

### Spheroid immunofluorescence

**FIGURE 10:**
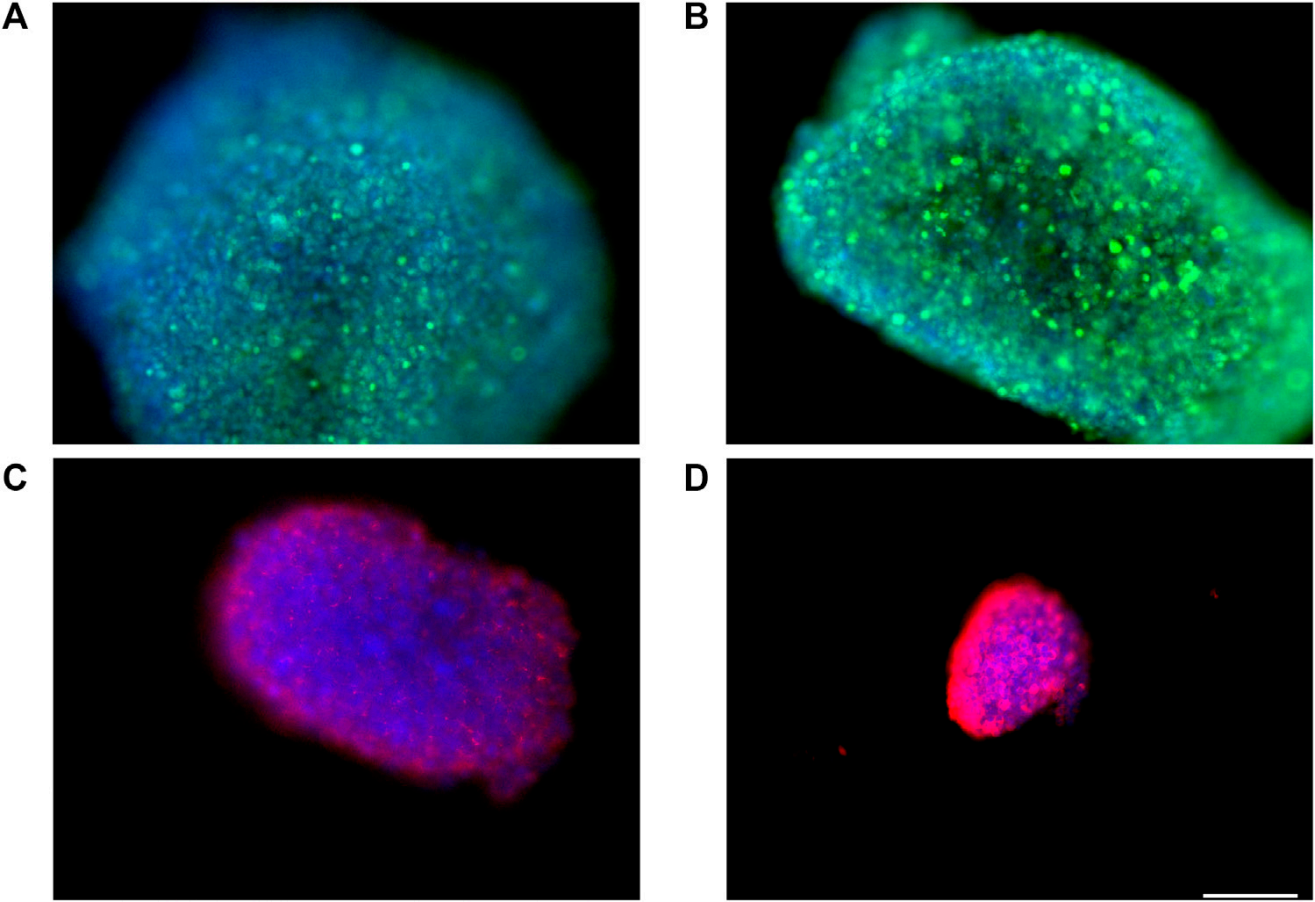
Representative image of spheroids obtained by culturing human neuroblastoma SH-SY5Y SH-SY5Y cells in the lower chamber of LB2/bEnd.3 cells. Spheroids were immunoreacted with Ki-67 antibody, **(**GFP, **A**,**B)**, ZO1 **(**RFP,**C),** and β-tubulin III **(**RFP,**D)**. Nuclei, DAPI. Images were acquired as Z stacks with the JuLI Stage fluorescence recorder, and the maximum intensity projection was shown. The scale bar was 250 µm.

3D SH-SY5Y were cultured in hanging drop culture and then transferred to LB1 to set the suitable flow condition. Once determined, different indirect immunofluorescence assays were performed to evaluate the presence of Ki-67, ZO-1, and β3-tubulin proteins. After 1 h and 45 min of incubation with appropriate antibodies, 3D SHSY5Y cells showed an obvious fluorescence signal for Ki-67 (Figure 10A) proliferation protein, demonstrating the proliferation activity of 3D SH-SY5Y cells in dynamic conditions. The presence of the tight junction protein ZO-1 shows the tight adhesion of cells under the flow (Figure 10B), while β3-tubulin shows physiological neural marker on enriched 3D SH-SY5Y (Figure 10C). Nuclei were stained with DAPI.

### gH625-lipoPACAP-Rho through a 3D dynamic *in vitro* blood–brain barrier millifluidic model

**FIGURE 11:**
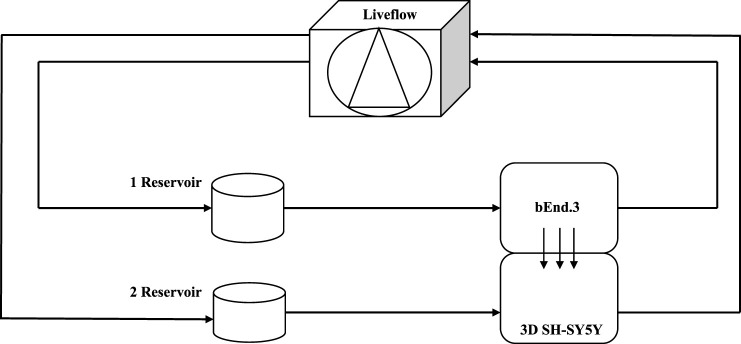
Schematic view of a dynamic bioreactor “Live Box 2 (LB2)” is composed by an upper chamber connected to a medium reservoir and to a Liveflow pump (250 μL/min) and a lower chamber connected to a medium reservoir and to a Liveflow pump (250 μL/min). In the upper chamber, bEnd.3 cells are seeded on the porous membrane, and there are neuroblastoma cells in the lower chamber enriched with neural portion (3D SH-SY5Y).

**FIGURE 12:**
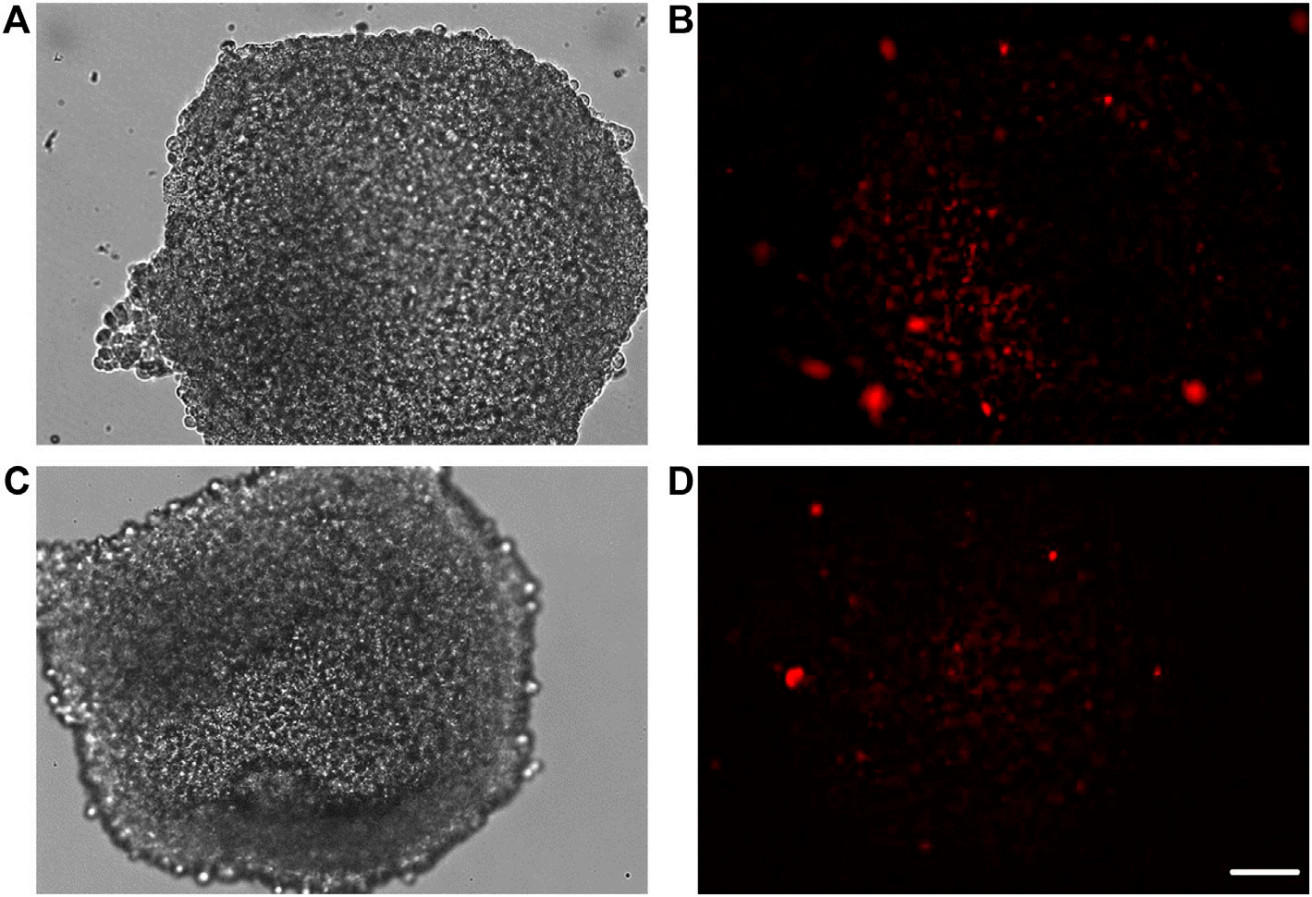
Representative images of spheroids obtained by culturing human neuroblastoma SH-SY5Y cells in the lower chamber of LB2/bEnd.3 cells **(A–D)**. After the passage of gH625-liposome PACAP-Rho, spheroids retain labeling for PACAP-Rho **(B–D)**. Images were acquired as Z stacks with the JuLI Stage fluorescence recorder and the maximum intensity projection was shown. The scale bar was 250 μm.

Once 3D SH-SY5Y cells were adapted to the flow in LB1, after 24 h, they were transferred in the lower chamber of LB2 containing bEnd.3 cells seeded in the upper chamber for 7 days. This 3D BBB set is useful to perform the passage of gH625-lipoPACAP-Rho in LB2 (Figure11). After 2 h of the injection of the gH625-lipoPACAP-Rho into the inlet tube of LB2, we can show Rho signal in 3D SH-SY5Y cells, demonstrating the passage of PACAP-Rho through the endothelial cells’ monolayer and the presence of this latter in 3D SH-SY5Y cells (Figure 12).

## Discussion

The BBB is a highly selective anatomical-functional structure. Its functions are due to complex anatomy, which does not allow the diffusion of many solutes from the interstitial fluid to the cerebral parenchyma. This allows the barrier to perform its main neuroprotective function against toxins and metabolites, but at the same time, many drugs are unable to cross the BBB and are pumped externally by an active transporter (Alyautdin et al., 2014). These pumps, while performing a protective action represent, at least in some cases, an obstacle in the treatment of neurodegenerative diseases (Alavijeh et al., 2005). There are numerous *in vitro* approaches that allow to study the BBB in order to find a valid pharmacological approach. However, many *in vitro* systems are far from the actual physiological condition (for a complete engineering-based comparison of static vs. dynamic models please refer to Giusti et al., 2014), so recently *in vitro* dynamic models have been developed to recreate biological barriers and to modulate the cell culture environment to simulate the situation *in vivo* as much as possible (Rouwkema et al., 2011). Among these, millifluidic bioreactors guarantee a continuous circulation of nutrients and a relevant cell density (Mattei et al., 2014; Giusti et al., 2017). They are characterized by the presence of a membrane that divides an upper and a lower chamber, each with an independent flow. The ability to recreate a BBB model *in vitro* and reproduce as closely as possible the physiological conditions, represents a crucial point for managing neurodegenerative diseases and for formulating a possible cure for them. In this regard, this study aimed to analyze the efficiency of gH625-liposome to deliver PACAP through a dynamic millifluidic bioreactor model of the BBB. We demonstrated that bEnd.3 cells, commonly used for BBB *in vitro* (Dos Santos Rodrigues et al., 2019), form an intact monolayer after 1 week, as permeability assay and the expression of junction protein analyses indicate. Furthermore, we showed that cells are not damaged, both morphologically and physiologically. Our results are in agreement with the available literature (Dos Santos Rodrigues et al., 2019), confirming the main ability of bEnd.3 cells to form an integral barrier. Moreover, these cells can be easily adapted on the porous membrane of the LB2 bioreactor transforming it in a BBB bioreactor without toxic damage. Then, we used this *in vitro* system to evaluate the amounts of PACAP able to cross the bEnd.3 layer, seeded on the porous membrane. After the gH625-lipoPACAP-Rho injection into the inlet of the superior chamber of the bioreactor we monitored, by spectrofluorimetry, the distribution of PACAP-Rho between chambers and endothelial layer, respectively. Showing the increase in the amount of PACAP in the lower chamber after 30 min compared to the non-functionalized lipo-PACAP-Rho, we can state that gH625 functionalization increases the efficiency of the process of the endothelial layer crossing. The long-lasting fluorescence associated with gH625lipo PACAP, compared to non-functionalized liposomes and the time course, indicates a fast and robust passage of PACAP-Rho through the cells when delivered by a functionalized nanodelivery system. This is the first report, as far as we know, showing the efficient delivery of PACAP in *in vitro* dynamic conditions through the endothelial cell layer. It is noteworthy that morphological analyses showed the lack of fluorescent signal in the endothelial layer before and after the passage of gH625-lipoPACAP-Rho, suggesting that PACAP was conveyed to the lower chamber through the endothelial cell layer without any PACAP being retained within bEnd3 cells. These results are in good agreement with the available literature reporting *in vitro* very low cell toxicity and *in vivo* good BBB crossing and uptake properties for gH625 (Falanga et al., 2011; Valiante et al., 2015). We demonstrated that PACAP reaches the dopaminergic compartment at physiological concentration, 10^−8^ M; this result is very remarkable since PACAP usually acts as a neuroprotective agent at this physiological concentration (Vaudry et al., 2009). Hence, PACAP can be effectively released by a functionalized nanodelivery system through the endothelial layer inside our fluid-dynamic system. Since a neuronal compartment is mandatory to realize a complex 3D dynamic *in vitro* BBB model with an endothelial cell line and a neuronal cell line, we used an LB2 with bEnd.3 cells seeded in the upper chamber and 3D SH-SY5YN in the lower chamber. Immunofluorescence experiments showed that fluid dynamic conditions have no appreciable influence on spheroids health, morphology, and organization. Finally, our delivery experimental data, demonstrating that PACAP once delivered through the endothelial layer reaches neuronal cells, and allows us to consider fluid dynamic cell culture conditions as a primary choice in drug delivery studies.

## Conclusion

This study demonstrated the ability of our nanodelivery system, made by functionalized liposomes and loaded with specific molecule to cross a fluid-dynamic model of the BBB *in vitro*. This study allows us to state that 1) our fluid dynamic BBB model is suitable for enhanced drug delivery studies; 2) gH625 increases the release efficiency of PACAP in our *in vitro* fluid dynamic model of the BBB. These findings represent an important step for further experimental investigations on PACAP administration as a therapeutic agent by the enhanced drug delivery system. Furthermore, the implementation of our BBB model in the routine lab cell culture procedure could be helpful to fit the 3R principles.

## Data Availability

The original contributions presented in the study are included in the article/Supplementary Material; further inquiries can be directed to the corresponding author.
